# The effect of intermittent pneumatic compression on deep-vein thrombosis and ventilation-free days in critically ill patients with heart failure

**DOI:** 10.1038/s41598-022-12336-9

**Published:** 2022-05-20

**Authors:** Hasan M. Al-Dorzi, Abdulaziz Al-Dawood, Fahad M. Al-Hameed, Karen E. A. Burns, Sangeeta Mehta, Jesna Jose, Sami Alsolamy, Sheryl Ann I. Abdukahil, Lara Y. Afesh, Mohammed S. Alshahrani, Yasser Mandourah, Ghaleb A. Almekhlafi, Mohammed Almaani, Ali Al Bshabshe, Simon Finfer, Zia Arshad, Imran Khalid, Yatin Mehta, Atul Gaur, Hassan Hawa, Hergen Buscher, Hani Lababidi, Abdulsalam Al Aithan, Yaseen M. Arabi

**Affiliations:** 1 Intensive Care Department, Ministry of National Guard Health Affairs, Riyadh, Kingdom of Saudi Arabia; 2 King Abdullah International Medical Research Center, Riyadh, Kingdom of Saudi Arabia; 3 College of Medicine, King Saud Bin Abdulaziz University for Health Sciences, Riyadh, Kingdom of Saudi Arabia; 4 Intensive Care Department, Ministry of National Guard Health Affairs, Jeddah, Kingdom of Saudi Arabia; 5 King Abdullah International Medical Research Center Jeddah, King Saud Bin Abdulaziz University for Health Sciences, Jeddah, Kingdom of Saudi Arabia; 6 Interdepartmental Division of Critical Care Medicine, University of Toronto, Toronto, Canada; 7 Unity Health Toronto - St Michael’s Hospital, Toronto, Canada; 8 Li Ka Shing Knowledge Institute, Toronto, Canada; 9 Department of Medicine, Medical Surgical ICU, Mount Sinai Hospital, University of Toronto, Toronto, Canada; 10 Department Biostatistics and Bioinformatics, King Abdullah International Medical Research Center, Riyadh, Kingdom of Saudi Arabia; 11 King Saud Bin Abdulaziz University for Health Sciences, Riyadh, Kingdom of Saudi Arabia; 12 Research Office, King Abdullah International Medical Research Center, Riyadh, Kingdom of Saudi Arabia; 13 Department of Emergency and Critical Care Medicine, College of Medicine, King Fahd Hospital of the University-Imam Abdulrahman Bin Faisal University, Dammam, Kingdom of Saudi Arabia; 14 Military Medical Services, Ministry of Defense, Riyadh, Kingdom of Saudi Arabia; 15 Department of Intensive Care Services, Prince Sultan Military Medical City, Riyadh, Kingdom of Saudi Arabia; 16 Department of Pulmonary and Critical Care Medicine, King Fahad Medical City, Riyadh, Kingdom of Saudi Arabia; 17 Department of Critical Care Medicine, Asir Central Hospital, King Khalid University, Abha, Kingdom of Saudi Arabia; 18 The George Institute for Global Health, University of New South Wales, Sydney, Australia; 19 Department of Anesthesiology and Critical Care, King George’s Medical University, Lucknow, India; 20 Critical Care Section, Department of Medicine, King Faisal Specialist Hospital and Research Center, Jeddah, Kingdom of Saudi Arabia; 21 Institute of Critical Care and Anaesthesiology, Medanta - The Medicity, Gurgaon, Haryana India; 22 Intensive Care Department, Gosford Hospital, Gosford, New South Wales Australia; 23 Critical Care Medicine Department, King Faisal Specialist Hospital and Research Centre, Riyadh, Kingdom of Saudi Arabia; 24 Department of Intensive Care Medicine, Centre for Applied Medical Research, St. Vincent’s Hospital, University of New South Wales, Sydney, Australia; 25 Intensive Care Division, Department of Medicine, King Abdulaziz Hospital, King Abdullah International Medical Research Center, King Saud Bin Abdulaziz University for Health Sciences, Al Ahsa, Kingdom of Saudi Arabia

**Keywords:** Cardiology, Diseases

## Abstract

There are contradictory data regarding the effect of intermittent pneumatic compression (IPC) on the incidence of deep-vein thrombosis (DVT) and heart failure (HF) decompensation in critically ill patients. This study evaluated the effect of adjunctive use of IPC on the rate of incident DVT and ventilation-free days among critically ill patients with HF. In this pre-specified secondary analysis of the PREVENT trial (N = 2003), we compared the effect of adjunctive IPC added to pharmacologic thromboprophylaxis (IPC group), with pharmacologic thromboprophylaxis alone (control group) in critically ill patients with HF. The presence of HF was determined by the treating teams according to local practices. Patients were stratified according to preserved (≥ 40%) versus reduced (< 40%) left ventricular ejection fraction, and by the New York Heart Association (NYHA) classification. The primary outcome was incident proximal lower-limb DVT, determined with twice weekly venous Doppler ultrasonography. As a co-primary outcome, we evaluated ventilation-free days as a surrogate for clinically important HF decompensation. Among 275 patients with HF, 18 (6.5%) patients had prevalent proximal lower-limb DVT (detected on trial day 1 to 3). Of 257 patients with no prevalent DVT, 11/125 (8.8%) patients in the IPC group developed incident proximal lower-limb DVT compared to 6/132 (4.5%) patients in the control group (relative risk, 1.94; 95% confidence interval, 0.74–5.08, *p* = 0.17). There was no significant difference in ventilator-free days between the IPC and control groups (median 21 days versus 25 days respectively, *p* = 0.17). The incidence of DVT with IPC versus control was not different across NYHA classes (*p* value for interaction = 0.18), nor across patients with reduced and preserved ejection fraction (*p* value for interaction = 0.15). Ventilator-free days with IPC versus control were also not different across NYHA classes nor across patients with reduced or preserved ejection fraction. In conclsuion, the use of adjunctive IPC compared with control was associated with similar rate of incident proximal lower-limb DVT and ventilator-free days in critically ill patients with HF.

Trial registration: The PREVENT trial is registered at ClinicalTrials.gov, ID: NCT02040103 (registered on 3 November 2013, https://clinicaltrials.gov/ct2/show/study/NCT02040103) and Current controlled trials, ID: ISRCTN44653506 (registered on 30 October 2013).

## Introduction

Heart failure (HF) is a major risk factor for venous thromboembolism (VTE), whether deep-vein thrombosis (DVT) or pulmonary embolism^1–5^. The prevalence of DVT among patients with HF has been reported to range from 4 to 26% and the prevalence of pulmonary embolism as high as 9.1%^6^. The risk of VTE increases as the left ventricular ejection fraction decreases and as the New York Heart Association (NYHA) functional class increases^2,7^. In acutely or critically ill medical patients, pharmacologic thromboprophylaxis is preferred over no pharmacologic prophylaxis^8^. This was based on evidence from multiple randomized trials showing that pharmacologic prophylaxis versus placebo was more effective^9–12^. In patients with HF, who constituted 34.1 to 51.7% of patients enrolled in these trials^9–11^, the reduction in VTE rates was by 26–59%^6,7,9–11,13^. Pharmacologic thromboprophylaxis is also preferred over mechanical prophylaxis in acutely or critically ill medical patients (very low certainty in the evidence)^8,14^. In at-risk hospitalized patients with a contraindication for pharmacologic thromboprophylaxis, mechanical prophylaxis with intermittent pneumatic compression (IPC)^15^ is recommended^8,16–22^. In addition, IPC has been recommended as an adjunct to pharmacologic prophylaxis in selected high-risk populations, including subgroups of critically ill patients^20,22^.

IPC devices are thought to prevent venous thrombi by increasing venous blood flow and reducing stasis in the leg veins^23^. As hospitalized patients with HF frequently have lower limb venous congestion and pulmonary edema, concerns exist regarding the use of IPC in this patient cohort due to the potential of worsening HF. IPC can augment venous return, and increase both central venous and pulmonary artery pressures^24^. These physiologic effects may theoretically exacerbate HF leading to the suggestion that IPC should not be used for thromboprophylaxis in patients with HF^25^.

Only a few small studies have assessed the hemodynamic effects of IPC in patients with HF—including small numbers of patients with heterogenous severity of HF and assessing short-term physiologic changes—and have not demonstrated HF decompensation^26–28^. In a study of 20 patients with HF monitored by pulmonary artery catheterization, there were no significant changes in any hemodynamic parameters and no clinical deterioration^26^. In 19 patients with left ventricular ejection fraction < 40% (mean 29%) and NYHA class II and III, thigh-length IPC did not exacerbate symptoms and transiently improved cardiac output, probably through an increase in stroke volume and a reduction in systemic vascular resistance^27^. No detrimental effect on diastolic cardiac function and no adverse clinical events were noted^27^. In 14 patients with HF (left ventricular ejection fraction < 40% and NYHA class II and III), IPC, which was activated only after intravenous diuretics and symptomatic improvement, did not lead to significant differences in blood pressure, central venous pressure, systemic vascular resistance or cardiac output^28^. Additionally, brain natriuretic peptide levels did not change^28^.

Studies evaluating the effectiveness of combined mechanical and pharmacologic prophylaxis versus pharmacologic prophylaxis alone among very high risk patient groups have been advocated^8^. As critically ill patients with HF have multiple risk factors for VTE, the adjunctive use of IPC with pharmacologic prophylaxis has an unknown but potentially additive effect on VTE prevention. However, whether IPC has clinically important adverse effects remains unknown. In this preplanned secondary analysis of the PREVENT trial, we tested the hypothesis that adjunctive IPC reduces the incidence of DVT among critically ill patients with HF without precipitating HF decompensation, or increasing mortality.

## Methods

### The PREVENT trial

The PREVENT trial (Pneumatic Compression for Preventing Venous Thromboembolism trial, Clinicaltrials.gov: NCT02040103 and Current controlled trials: ISRCTN44653506)^29,30^ evaluated whether adjunctive IPC combined with pharmacologic thromboprophylaxis with unfractionated heparin or low-molecular-weight heparin compared to pharmacologic thromboprophylaxis alone, reduced incident proximal lower-limb DVT. The trial was conducted at 20 sites in Saudi Arabia, Canada, Australia, and India. We enrolled adult medical, surgical, or trauma ICU patients who weighed at least 45 kg, were expected to stay in ICU for at least 72 h and were eligible for pharmacologic thromboprophylaxis with either unfractionated heparin or low-molecular-weight heparin. Twice-weekly lower-limb ultrasonography was performed until ICU discharge, death, full mobility, or 28 days after enrollment, whichever occurred first. The trial demonstrated that adjunctive IPC did not result in a reduction in incident proximal leg DVT compared with pharmacologic prophylaxis^31^. All methods were carried out in accordance with relevant guidelines and regulations including Good Clinical Practice^32^.

### Patients

In the current study, we performed an a priori determined analysis^29,30^ of critically ill patients with HF. The presence of HF was determined by the treating teams according to local practices, which was based on the reported symptoms and signs that were typical of congestion with compatible findings on chest radiography, elevated natriuretic peptide biomarkers and/or cardiac imaging^33,34^. We further categorized patients based on NYHA functional class^35^ and left ventricular ejection fraction. NYHA class (I to IV) was determined by reviewing the reported symptoms and activity level before enrollment in the trial. The left ventricular ejection fraction (≥ 40% versus < 40%) was obtained from echocardiography performed before enrollment in the trial. This ejection fraction cutoff has been used in several clinical trials to define HF with reduced (< 40%) versus preserved ejection fraction (≥ 40%)^33,34,36^.

### Intervention and co-interventions

In patients who were randomized to IPC, the device was applied to both lower limbs for at least 18 h per day, with the sleeves removed every 8 h for skin inspection and care^31^. The study protocol prioritized the use of sequential compression devices (multi-chamber cuffs) and thigh-length sleeves when available but permitted the use of non-sequential devices (single-chamber cuffs) and knee-length sleeves. IPC use was discontinued for suspected or confirmed DVT, pulmonary embolism, leg ulcer, or ischemia; and at the discretion of the treating team, for palliation, full mobility, ICU discharge, or at study day 28^31^. In the control group, IPC was permitted only during interruption of pharmacologic thromboprophylaxis. Graduated compression stockings were not permitted in either group. Other aspects of patient management were at the discretion of the treating team, including post-randomization prescription of systemic anticoagulation and anti-platelet agents, HF management, and investigation for pulmonary embolism^31^.

### Measurements

Bilateral proximal lower-limb venous ultrasound was performed within 48 h of randomization, then twice weekly and on clinical suspicion of DVT by certified ultrasonographers^31^. The venous system was assessed for compressibility at 1-cm intervals at the following locations: common femoral vein, proximal superficial femoral vein, mid superficial femoral vein, distal superficial femoral vein, popliteal vein and venous trifurcation^31^. The ultrasound studies were interpreted by radiologists who were unaware of the patient’s treatment assignment. Proximal DVT was defined as partial or complete incompressibility of a venous segment in any site. Examination of the distal leg veins (peroneal, posterior tibial, anterior tibial, and muscular veins) was performed based on local hospital practices^31^.

### Data collection

We documented demographic information, Acute Physiologic Assessment and Chronic Health Evaluation II (APACHE II) score at ICU admission, VTE risk factors before ICU admission (hospitalization in the preceding 3 months for any reason, paralysis or immobilization of a lower or upper extremity related to stroke or injury prior to hospital admission, active malignancy, recent surgery, acute stroke, trauma, personal history of VTE, family history of VTE, known thrombophilia, post-partum state within 3 months, and estrogen therapy), and specific information regarding HF including NYHA functional classification (I to IV), and left ventricular ejection fraction of < 40% or ≥ 40%. We collected data on the intervention (IPC type and duration) and co-interventions (including agent of pharmacologic prophylaxis, therapeutic anticoagulation for reasons other than VTE, use of organ support (vasopressors, mechanical ventilation, and renal replacement therapy), location of central venous catheters, use of antiplatelets and statins. We also noted the diagnostic workup for pulmonary embolism and non-lower-limb venous thrombosis, which were requested at the discretion of the treating team.

The primary outcomes were incident proximal lower-limb DVT (diagnosed after day 3) and ventilator-free days. We considered ventilator-free days to be a surrogate for decompensation of HF. Secondary outcomes included prevalent lower-limb DVT (detected on trial days 1 to 3), incident distal lower-limb thrombosis, non-lower-limb venous thrombosis, acute pulmonary embolism, mechanical ventilation duration, vasopressor-free days, days to incident lower-limb DVT, ICU and hospital mortality and mortality at 28 and 90 days.

### Statistical analysis

We compared the baseline characteristics, intervention, and co-interventions between the IPC and control groups. We used Student’s t-test or the Mann–Whitney U test for continuous variables based on normality assumption and the chi-square test or Fisher’s exact test for categorical variables as appropriate. The effect of IPC versus no IPC on binary categorical outcomes was presented as a relative risk with 95% confidence interval (CI). Kaplan–Meier curves were used to compare the freedom from incident lower-limb DVT within the first 28 days in ICU and 90-day survival. The log rank test was used to compare the two groups.

We assessed incident proximal lower-limb DVT, ventilator-free days and 90-day mortality in selected subgroups and reported the results of tests of interactions. The subgroups were unfractionated heparin and low-molecular-weight heparin for thromboprophylaxis, BMI < 30 kg/m^2^ and BMI ≥ 30 kg/m^2^, ejection fraction of < 40% and ≥ 40%, femoral central venous catheter and no femoral central venous catheter at the time of enrollment, mechanical ventilation and no mechanical ventilation, NYHA classes I to IV, and receipt of vasopressors and no vasopressors^29^. A *p* value < 0.05 was considered statistically significant. All analyses were conducted using SAS software, version 9.4 (SAS Institute, Cary, NC, USA).

### Ethical approval and consent to participate

Ethics approval was obtained from the Institutional Review Board of the Ministry of National Guard Health Affairs, Riyadh Saudi Arabia (primary site) and the Institutional Review Boards of the participating centers. Informed consents were obtained from enrolled patients.

## Results

### Characteristics at baseline

Of the 2003 patients in the PREVENT trial, 275 had HF, as assessed by the clinical teams. Of these, 133 patients were randomly assigned to the IPC (intervention) group and 142 to the control group. The two groups had similar baseline characteristics: age 70 ± 14 years for the IPC group and 68 ± 16 years for the control group (*p* = 0.31); APACHE II score 23.5 ± 7.3 and 22.9 ± 6.9, respectively (*p* = 0.53); presence of at least one VTE risk factor 60.2% and 68.3%, respectively (*p* = 0.20); receipt of mechanical ventilation in 69.9% and 64.1%, respectively (*p* = 0.30), vasopressor therapy for 48.9% and 43.7%, respectively (*p* = 0.39) (Table 1). Overall, 140 (50.9%) patients in the two groups had NYHA class III or IV symptoms and 105 (38.2%) had left ventricular ejection fraction < 40% documented on echocardiography. Data about ejection fraction were not available for 53 patients because they did not have an echocardiogram performed.

**Table 1:** Baseline characteristics of patients with heart failure who were randomized to intermittent pneumatic compression with pharmacologic thromboprophylaxis (IPC group) or pharmacologic thromboprophylaxis alone (control group).

	IPC group (N = 133)	Control group (N = 142)	*p* value
Age (years), mean (SD)	70.0 ± 14.4	68.5 ± 15.5	0.31
Male sex—n (%)	74 (55.6)	80 (56.3)	0.91
Body mass index (kg/m^2^)—mean (SD)	30.4 ± 9.4	30.1 ± 8.5	0.91
**Location prior to ICU admission—n (%)**			
Emergency room	70 (52.6)	80 (56.3)	0.62
Hospital ward	47 (35.3)	44 (31.0)	
Operating room	6 (4.5)	9 (6.3)	
Other hospital (ICU or ward)	8 (6.0)	9 (6.3)	
Other	2 (1.5)	0 (0)	
APACHE II score—mean (SD)	23.5 ± 7.3	22.9 ± 6.9	0.53
**Admission category–n (%)**			
Medical	124 (93.2)	122 (85.9)	0.09
Post-operative unrelated to trauma	6 (4.5)	16 (11.3)	
Trauma-related	3 (2.3)	4 (2.8)	
**Chronic health illnesses–n (%)**			
None	10 (7.5)	11 (7.7)	0.79
Chronic respiratory disease	36 (27.1)	40 (28.2)	
End-stage renal disease	34 (25.6)	29 (20.4)	
Immunosuppression	10 (7.5)	8 (5.6)	
Chronic liver disease	8 (6.0)	5 (3.5)	
**Heart failure defined by New York Heart Association Functional Classification—n (%)**			
Class I	20 (15.0)	26 (18.3)	0.82
Class II	41/132 (31.1)	47 (33.1)	
Class III	55/132 (41.7)	52 (36.6)	
Class IV	16/132 (12.1)	17 (12.0)	
**Left ventricular ejection fraction—n (%)**			
Not available	30 (22.6)	23 (16.2)	0.34
≥ 40%	52 (39.1)	65 (45.8)	
< 40%	51 (38.3)	54 (38.0)	
**Pre-ICU VTE risk factors—n (%)**			
None	53 (39.8)	45 (31.7)	0.14
Hospitalization in the past 3 months for any reason (excluding candidate hospital admission)	46 (34.6)	49 (34.5)	
Paralysis or immobilization of a lower or upper extremity related to stroke or injury prior to this hospital admission	16 (12.0)	21 (14.8)	
Active malignancy (treatment within past 6 months or palliation)	7 (5.3)	14 (9.9)	
Recent surgery (in the last 48 h)	5 (3.8)	11 (7.7)	
Acute stroke (this hospital admission)	12 (9.0)	4 (2.8)	
Trauma	2 (1.5)	4 (2.8)	
History of malignancy (past 5 years; other than non-melanoma skin cancer)	4 (3.0)	4 (2.8)	
Personal history of VTE	2 (1.5)	0 (0)	
Family history of VTE	0 (0)	1 (0.7)	
Known thrombophilia	0 (0)	0 (0)	
Post-partum (within 3 months)	0 (0)	1 (0.7)	
Estrogen therapy	0 (0)	0 (0)	
Others	1 (0.8)	4 (2.8)	
**Laboratory results prior to randomization**			
INR—mean (SD)	1.3 ± 0.7	1.2 ± 0.3	0.24
Creatinine (µmol/L)—median (Q1, Q3)	148 (83, 261)	139.5 (90, 231)	0.55
Platelets (10^9^/L)—mean (SD)	245.7 ± 125.8	239.1 ± 131.7	0.53
PTT (sec)—mean (SD)	33.3 ± 10.4	33.6 ± 9.8	0.33
Hemoglobin (g/L)—mean (SD)	111.2 ± 81.4	100.3 ± 35.5	0.64
Femoral central venous line, n (%)	29 (21.8)	28 (19.7)	0.67
**Organ support—n (%)**			
Mechanical ventilation	93 (69.9)	91 (64.1)	0.30
Vasopressors	65 (48.9)	62 (43.7)	0.39
**Pharmacologic thromboprophylaxis at enrollment—n (%)**			
Unfractionated heparin	104 (78.2)	111 (78.2)	1.0
Low molecular weight heparin	29 (21.8)	31 (21.8)	
Pneumatic compression prior to randomization—n (%)	24 (18.0)	13 (9.2)	0.03

*APACHE* Acute physiology and chronic health evaluation, *ICU* Intensive care unit, *INR* International normalized ratio, *IPC* Intermittent pneumatic compression, *PTT* Partial thromboplastin time, *Q1* First quartile, *Q3* Third quartile, *SD* Standard deviation, *VTE* Venous thromboembolism.

### Intervention and co-interventions

IPC was applied mainly using knee-length sleeves (120 of 133 patients [90.2%] in the IPC group). It was applied for at least one day in 131/133 (98.5%) patients in the IPC group for a median duration of 22 h per day.

The use of pharmacologic thromboprophylaxis did not differ between the two groups at the time of randomization and during the trial, with approximately 80% receiving unfractionated heparin in the two groups at the time of randomization. Therapeutic anticoagulation was used after randomization for reasons other than VTE in 15/133 (11.3%) patients in the IPC group and 15/143 (10.6%) patients in the control group (*p* = 0.85). The use of antiplatelet therapy and statins was similar in the two groups. Moreover, there were no differences in the frequency and location of central venous catheters. Diagnostic investigations for the different forms of VTE were similar in both groups. Other cointerventions are shown in Table 2 and were generally similar in the two groups except for more frequent use of continuous renal replacement therapy in the IPC group compared with the control group (29 patients [21.8%] compared with 17 patients [12.0%]).

**Table 2:** ICU interventions and co-interventions in patients with heart failure who were randomized to intermittent pneumatic compression with pharmacologic thromboprophylaxis (IPC group) or pharmacologic thromboprophylaxis alone (control group).

	IPC group (N = 133)	Control group (N = 142)	*p* value
Median no. of days of the trial intervention (Q1, Q3)	7 (4,15)	7 (4,12)	0.65
**Use of pneumatic compression**			
Patients receiving pneumatic compression at least for one day, n (%)	131 (98.5)	13 (9.2)	< 0.0001
Daily duration of pneumatic compression (h)—median (Q1, Q3)	22 (21, 22)	0 (0, 0)	< 0.0001
Use of foot pumps—n (%)	12 (9.0)	1 (0.7)	0.001
Knee-length	120 (90.2)	13 (9.2)	< 0.0001
Thigh-length	11 (8.3)	0 (0)	
**Organ support—n (%)**			
Mechanical ventilation	98 (73.7)	98 (69.0)	0.39
Vasopressors	80 (60.2)	71 (50.0)	0.09
**Renal replacement therapy**			
Continuous renal replacement	29 (21.8)	17 (12.0)	0.03
Intermittent dialysis	8 (6.0)	11 (7.7)	0.57
Peritoneal dialysis	0	1 (0.7)	1.0
**Pharmacologic prophylaxis—n (%)**			
Prophylactic UFH	110 (82.7)	114 (80.3)	0.61
Prophylactic LMWH	29 (21.8)	34 (23.9)	0.67
Therapeutic anticoagulation after randomization for reasons other than venous thromboembolism—n (%)	15 (11.3)	15 (10.6)	0.85
Duration (days)—median	5 (2, 11)	3 (2, 5)	0.25
Warfarin—n (%)	1 (0.8)	2 (1.4)	1.0
Other oral anticoagulants—n (%)	2 (1.5)	2 (1.4)	1.0
Argatroban—n (%)	0	1 (0.7)	1.0
**Antiplatelet therapy—n (%)**			
Aspirin	75 (56.4)	74 (52.1)	0.48
Clopidogrel	29 (21.8)	31 (21.8)	1.0
Statin therpay—n (%)	72 (54.1)	74 (52.1)	0.74
Central venous catheters*—n (%)	92 (69.2)	97 (68.3)	0.88
Femoral central venous catheters– n (%)	43 (32.3)	39 (27.5)	0.38
Jugular or subclavian	74 (55.6)	74 (52.1)	0.56
Peripherally inserted central catheter	19 (14.3)	15 (10.6)	0.35
None	41 (30.8)	45 (31.7)	0.88
**Diagnostic imaging**			
Lower limb ultrasound per patient, median (Q1, Q3)	2 (1, 4)	2 (1, 4)	0.54
Ultrasonography for upper limb and neck to evaluate for thrombosis—n (%)	5 (3.8)	4 (2.8)	0.74
Chest CT for PE—n (%)	5 (3.8)	7 (4.9)	0.64
Ventilation/perfusion scan of the lungs—n (%)	0	0	
Abdominal CT—n (%)	9 (6.8)	5 (3.5)	0.22
Transthoracic echocardiograms—n (%)	19 (14.3)	24 (16.9)	0.55
Transesophageal echocardiograms—n (%)	1 (0.8)	1 (0.7)	1.0

*CT* Computed tomography, *IPC* Intermittent pneumatic compression, *LMWH* Low molecular weight heparin, *PE* Pulmonary embolism, *Q1* First quartile, *Q3* Third quartile, *UFH* Unfractionated heparin.

### Outcomes

Prevalent proximal lower-limb DVT was observed in 8/133 (6.0%) patients in the IPC group and 10/142 (7.0%) patients in the control group (*p* = 0.73). Among patients with no prevalent DVT, 11/125 (8.8%) patients in the IPC group developed incident proximal lower-limb DVT compared to 6/132 (4.5%) patients in the control group (relative risk, 1.94; 95% confidence interval, 0.74–5.08, *p* = 0.17) (Table 3). The rate of VTE consisting of prevalent and incident lower-limb DVT and pulmonary embolism was similar in both groups (17.3% in IPC group and 12.7% in control group, *p* = 0.28).

**Table 3:** Outcomes of patients with heart failure who were randomized to intermittent pneumatic compression with pharmacologic thromboprophylaxis (IPC group) or pharmacologic thromboprophylaxis alone (control group).

	IPC group (N = 133)	Control group (N = 142)	Relative risk, (95% CI)	*p* value
Incident proximal lower-limb DVT—n/N (%)	11/125 (8.8)	6/132 (4.5)	1.94 (0.74, 5.08)	0.17
**Venous thromboembolism secondary outcomes**				
Prevalent proximal lower limb DVT—n/N (%)	8/133 (6.0)	10/142 (7.0)	0.85 (0.35, 2.10)	0.73
All incident DVT (proximal and distal) —n/N (%)	14/125 (11.2)	8/ 132 (6.1)	1.85 (0.80, 4.25)	0.14
All lower limb DVT (proximal and distal, incident and prevalent)—n/N (%)	23/133 (17.3)	18/ 142 (12.7)	1.36 (0.77, 2.41)	0.28
PE—n (%)	0 (0.0)	1 (0.7)	–	0.33
Venous thromboembolism (all lower limb DVT and PE)—n/N (%)	23/133 (17.3)	19/ 142 (13.4)	1.29 (0.74, 2.26)	0.37
Non-lower limb venous thrombosis—n/N (%)	2/133 (1.5)	1/142 (0.7)	2.14 (0.20, 23.28)	0.52
Mechanical ventilation-free days—median (Q1, Q3)	21 (5, 27)	25 (10, 28)		0.17
Duration of mechanical ventilation (days)—median (Q1, Q3)	7 (3, 14)	6 (2, 11)		0.40
Duration of vasopressor use (days)—median (Q1, Q3)	3 (2, 8)	3 (2, 5)		0.67
Vasopressor-free days—median Q1, Q3	26 (17, 28)	27 (23, 28)		0.08
ICU length of stay (days)—median (Q1, Q3)	9 (5, 22)	8 (5, 16)		0.47
ICU-free days—median (Q1, Q3)	15 (0, 22)	18 (0, 23)		0.21
Hospital length of stay (days)—median (Q1, Q3)	24 (12, 48)	20 (11, 37)		0.18
ICU mortality—n (%)	26 (19.5)	21 (14.8)	1.32 (0.78, 2.23)	0.29
28-day mortality—n (%)	27 (20.3)	23 (16.2)	1.25 (0.76, 2.07)	0.38
Hospital mortality—n (%)	48 (36.1)	45 (31.7)	1.14 (0.82, 1.59)	0.44
90-day mortality—n (%)	43 (32.3)	43 (30.3)	1.07 (0.75, 1.52)	0.71
Composite endpoint of lower-limb DVT, PE and 28-day mortality—n (%)	46 (34.6)	37 (26.1)	1.33 (0.92, 1.91)	0.12

*CI* Confidence interval, *DVT* Deep vein thrombosis, *ICU* Intensive care unit, *IPC* Intermittent pneumatic compression, *Q1* First quartile, *Q3* Third quartile, *PE* Pulmonary embolism.

There was no significant difference in ventilator-free days between the IPC and control groups (21 versus 25 days respectively; *p* = 0.17). Additionally, there were no differences in pulmonary embolism rate, days to proximal lower-limb DVT, mechanical ventilation duration, and mortality between the IPC and control groups (Table 3).

**Figure 1:**
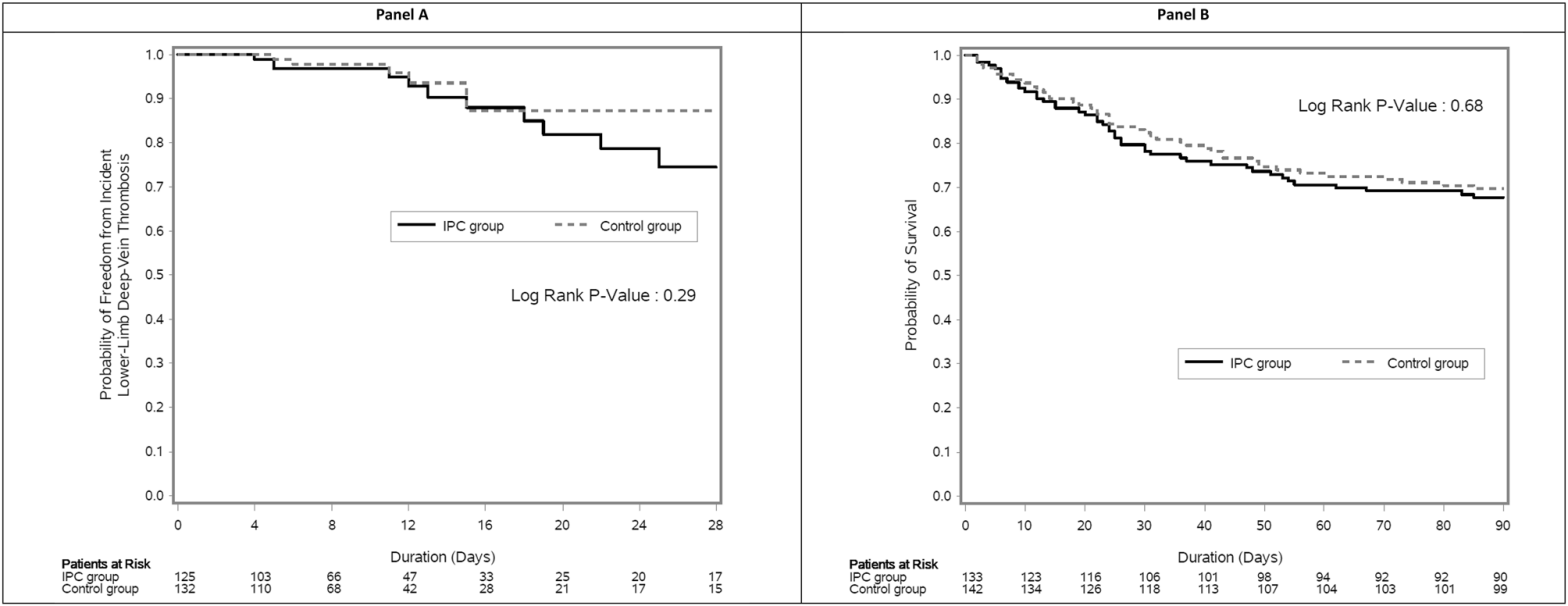
Kaplan–Meier curves for the freedom from incident lower-limb deep-vein thrombosis within 28 days (Panel **A**) and for 90-day survival (Panel **B**) in patients with HF randomized to receive intermittent pneumatic compression with pharmacologic thromboprophylaxis (IPC group) or pharmacologic thromboprophylaxis alone (control group). The log rank test was used to compare the two groups.

The Kaplan–Meier curves showed no differences in the freedom from incident lower-limb DVT within 28 days (Fig. 1A) and 90-day survival (Fig. 1B) between the IPC and control groups.

**Figure 2:**
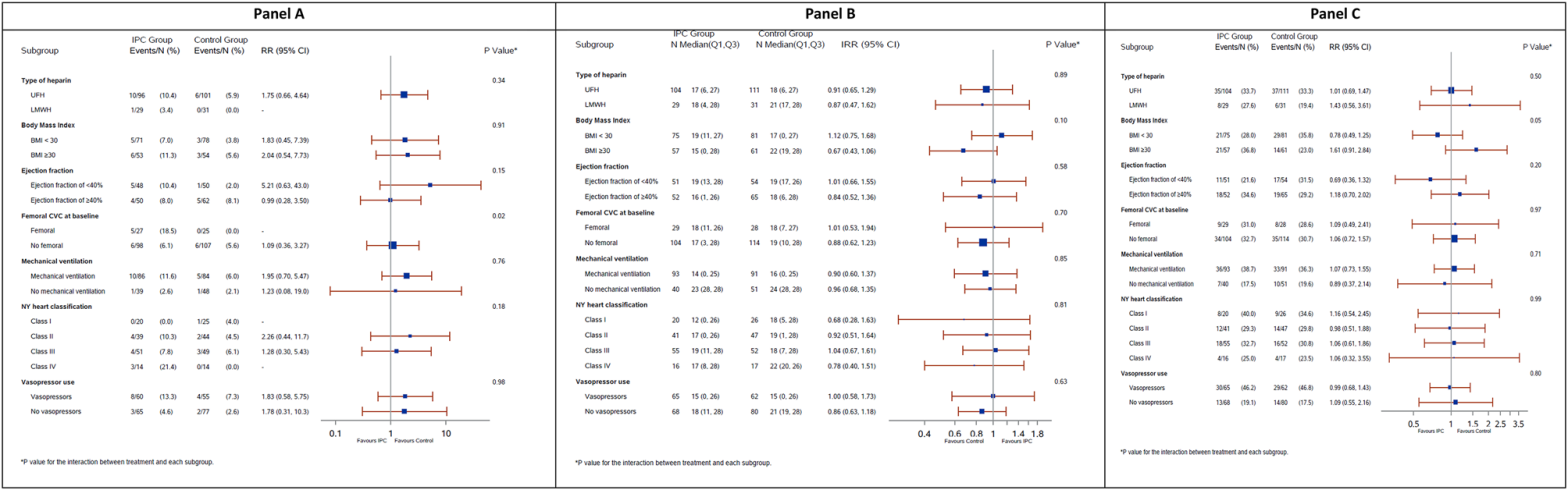
Forest plots showing incident lower-limb deep-vein thrombosis (Panel **A**), ventilator-free days (Panel **B**) and 90-day mortality (Panel **C**) in selected subgroups of patients with heart failure who were randomized to intermittent pneumatic compression with pharmacologic thromboprophylaxis (IPC group) or pharmacologic thromboprophylaxis alone (control group). The relative risk (RR) is reported for incident lower-limb deep-vein thrombosis and 90-day mortality. The incident rate ratio is reported for the ventilator-free days. The *p* value for the interaction between treatment and each subgroup is also reported.

The occurrence of incident lower-limb DVT with IPC versus control was not different across NYHA classes (*p* value for interaction = 0.18), or between patients with left ventricular ejection fraction of < 40% and ≥ 40% (*p* value for interaction = 0.15, Fig. 2, Panel A). Similarly, ventilator-free days and 90-day mortality with IPC versus control was not different across NYHA classes (Fig. 2, Panel B), or between patients with left ventricular ejection fraction of < 40% and ≥ 40% (Fig. 2, Panel C).

## Discussion

The main findings of this study were that incident lower-limb DVT and ventilator-free days were not different between HF patients who received adjunctive IPC and those who did not. Moreover, the two groups were not different in secondary outcomes including 90-day mortality. There was no heterogeneity in the effect of incidence of DVT, ventilation-free days or 90-day mortality across any of subgroups, including different severities of HF.

VTE rates in hospitalized patients with HF range between 4 and 26%^6^. The largest study that evaluated pharmacologic prophylaxis in patients with HF randomized 3,706 acutely ill medical patients to subcutaneous dalteparin 5000 IU daily or placebo for 14 days^10^. Data on mechanical prophylaxis were not reported^10^. More than 50% (n = 1905) of the enrolled patients had acute congestive HF (NYHA class III or IV)^10^; in these patients the incidence of VTE, defined as the combination of symptomatic DVT, symptomatic pulmonary embolism, and asymptomatic proximal DVT, was 33/781 (4.2%) in the placebo group and 25/814 (3.1%) in the dalteparin group (relative risk, 0.73; 95% CI, 0.44–1.21)^13^. The patients in this study were not critically ill^13^, which might have made them at lower VTE risk compared to the patients in the current study, in which incident lower-limb DVT occurred in 17/257 (6.6%) critically ill patients with HF. Hence, evaluating combined mechanical and pharmacologic prophylaxis versus pharmacologic prophylaxis is justified.

IPC prevents DVT mainly by augmenting venous blood flow and reducing hypercoagulability as it stimulates the fibrinolytic activity of vessel walls^37,38^. The effectiveness of IPC may be negatively altered by the presence of lower limb edema. We found that IPC as an adjunct to pharmacologic thromboprophylaxis was not associated with a reduction in incident VTE, however our trial was not powered to detect a difference across varying severities of HF.

IPC may increase venous blood return and theoretically exacerbate pulmonary edema, which may worsen the outcomes of affected patients. In healthy volunteers, IPC has been shown to increase cardiac output by increasing venous return^39^. In healthy patients undergoing elective Cesarean section under spinal anesthesia, IPC caused less hemodynamic instability as the decrease in mean arterial pressure by > 20% occurred less frequently in those who had IPC (13/25 [52%] patients versus 23/25 [92%] in the control group)^40^. In a study of 18 healthy patients admitted to ICU postoperatively, venous return increased with no change in cardiac output during the application of IPC^41^. Three small randomized controlled trials observed no HF decompensation with IPC use^26–28^, however these patients were not critically ill and these studies primarily assessed short-term physiologic changes^26–28^. In the current study, IPC use was associated with similar ventilator-free days and duration of mechanical ventilation, suggesting that they may not lead to HF exacerbation or have any effect on clinically important outcomes.

The strengths of our study include the multicenter prospective data collection and the predetermined subgroup analysis of a randomized controlled trial. The study is limited by a relatively small sample size, leading to inadequate power to demonstrate statistically significant differences in outcomes. However, data on IPC in critically ill patients with HF are scarce. Other limitations include the classification of HF based on the assessment by clinicians, and the lack of evaluation of direct measures of HF decompensation after the use of IPC, such as the occurrence of pulmonary edema, brain natriuretic peptide levels, and worsening of hypoxia. Our study does not address the question of whether IPC reduces the incidence of DVT in patients with HF who are not receiving pharmacologic prophylaxis.

## Conclusions

This predetermined analysis of the PREVENT randomized trial found that among patients with HF who received pharmacologic thromboprophylaxis, the use of IPC versus no IPC was associated with similar rate of incident proximal lower-limb DVT. Moreover, ventilator-free days were similar between groups, suggesting that IPC use may not lead to HF decompensation.

## Supplementary Information

Supplementary Information.

## Data Availability

The datasets used and/or analyzed during the current study are available from the corresponding author on reasonable request.

## Abbreviations

APACHE: Acute physiology and chronic health evaluation
CI: Confidence interval
DVT: Deep-vein thrombosis
HF: Heart failure
ICU: Intensive care unit
IPC: Intermittent pneumatic compression
NYHA: New York Heart Association
RR: Relative risk
VTE: Venous thromboembolism
