# Supplementary material for: The effect of intermittent pneumatic compression on deep-vein thrombosis and ventilation-free days in critically ill patients with heart failure
Source: Sci Rep. 2022 May 20;12:8519. doi: 10.1038/s41598-022-12336-9 (PMC9122920; doi:10.1038/s41598-022-12336-9)
Supplement: Supplementary file 1 — Supplementary Information. [file 41598_2022_12336_MOESM1_ESM.docx]

**Supplement**

| **Management Committee** | Dr. Yaseen M. **Arabi**  Dr. Abdulaziz **Al-Dawood**  Dr. Sami J. **Alsolamy**  Sheryl Ann I. **Abdukahil**  Lara Y **Afesh**  Jesna **Jose** |
| --- | --- |
| **Writing Committee** | Dr. Yaseen M. **Arabi**  Dr. Hasan **Al-Dorzi**  Sheryl Ann I. **Abdukahil**  Jesna **Jose**  Dr. Karen EA **Burns**  Dr. Sangeeta **Mehta**  Prof. Simon **Finfer** |
| **Data Monitoring Committee** | Prof. Kathryn **Rowan** (Chair)  Dr. Lehana **Thabane**  Dr. David **Garcia** |

**The Saudi Critical Care Trials Group**

| **Collaborators (*Sites are in alphabetical order)*** | |
| --- | --- |
| **Saudi Arabia** | |
| **Asir Central Hospital, Abha** | Dr. Ali **Al Bshabshe**  Dr. Abdulmoniem **Albahar**  Dr. Ali **Alamri**  Bincy **Mathew**  Nora **Assiri**  Dr. Ausama Omar **Ismaeil** |
| **King Abdulaziz Hospital, Ahsa** | Dr. Abdulsalam **Al Aithan**  Shahinaz **Bashir**  Dr. Syed **Hassan**  Jecel **Natavio**  Priscilla **Guiang**  Minda **Baisas** |
| **King Abdulaziz Medical City, Jeddah** | Dr. Fahad **Al-Hameed**  Dr. Gulam **Rasool**  Dr. Jalal **Rifai**  Ali S. **Mohamed**  Ohoud **Al Orabi**  Ferdos **Alahmary** |
| **King Abdulaziz Medical City, Riyadh** | Dr. Yaseen M. **Arabi**  Dr. Abdulaziz **Al-Dawood**  Dr. Sami J. **Alsolamy**  Dr. Mohamed **Hegazy**  Dr. Maamoun **Dbsawy**  Sheryl Ann I. **Abdukahil**  Lara Y **Afesh**  Jesna **Jose**  Dr. Ghassan **Al-Maziad**  Dr. Musharaf **Sadat**  Eman **Al Qasim**  Ahmad **Deeb**  Muhammed **Rafique**  Aron **Toledo**  Felwa **bin Humaid**  Amjad **Alaskar**  Ahmad **Al Khalaf**  Ahmed **Kanfer**  Albatole **Gorban**  Helen **Batin**  Mohammed **Al Shehri** |
| **King Fahad Medical City** | Dr. Mohammed **Almaani**  Dr. Hani **Lababidi**  Dr. Husain **Abdulmuthalib**  Pendo **Ntinika**  Emelinda **Ramos**  Ibrahim **AlEidan**  Nona **Bacani**  Dr. Ahmad **Al Jefri** |
| **King Fahd Hospital of the University, Imam Abdulrahman Bin Faisal University, Dammam** | Dr. Mohammed S **Alshahrani**  Laila Perlas **Asonto**  Kathrina **Libunao-de Loyola**  Charlene **Mapusao**  Dr. Mohammed **ElGalhoumy**  Dr. Ahmed Ali Abdl-Hali **Hassan**  Esperanza **Dipasupil**  Norma **Toledo** |
| **King Faisal Specialist Hospital and Research Center, Jeddah** | Dr. Imran **Khalid**  Dr. Ismael **Qushmaq**  Maryam **Imran**  Manahil **Imran**  Lenith **Salazar**  Nouf **Sulimani**  Jessel **Teves**  Johanna **Vega**  Joan **Ferrer** |
| **King Faisal Specialist Hospital and Research Center, Riyadh** | Dr. Hassan **Hawa**  Dr. Khalid **Maghrabi**  Dr. Mohammad **Hijazi**  Dr. Musaab **Abdelhai**  Ellen Joy **Pagunsan**  Marketa **Vinklerova**  Dr. Salahudin **Elnaas**  Muna **Al Zahrani**  Manal **Mustafa**  Lamya **Al AbdulAziz**  Nouf **Al Amri** |
| **Prince Sultan Military Medical City** | Dr. Ghaleb A. **Almekhlafi**  Dr. Yasser **Mandourah**  Dr. Sahar **Hassan**  Dr. Emad **Al Amodi**  Dr. Osama **Elfaki**  Dr. Ahmad **Alenazy**  Dr. Dina Al **Sufiani**  Dr. Bander **Alanazi**  Kholoud **Alharbi**  Shatha **Awad**  Ma. Raylin Cubio **Cabal**  Jean S. **Valerio**  Dr. Mohammed **Alkhader**  Esperanza **Bautista** |
| **Canada** | |
| **Mount Sinai Hospital** | Dr. Sangeeta **Mehta**  Sumesh **Shah**  Erik **Tamberg**  Crystal **Angaran**  Carlos **Arrazola**  Ashley **Briggs**  Angela **Calabrese**  Lyndsey **Cameron**  Erin **Cham**  Doreen **Chu**  Saerom **Chung**  Krista **Colley**  Ann **Downey**  Amy **He**  Kelli **Jackson**  Heather **Macdonald**  Oleksander **Malyarchuk**  Mirela **Papadhima**  Jamie **Song**  Anjuli **Sookoo**  Melissa **Wong**  Qiang **Zhang**  Tingting **Zhang**  Michelle **Zhen**  Ning **Zhu** |
| **Saint Boniface General Hospital** | Dr. Ryan **Zarychanski**  Nicole **Marten**  Dr. Justin **Lys** |
| **Saint Michael’s Hospital** | Dr. Karen EA **Burns**  Dr. Jan **Friedrich**  Dr. Andrew **Baker**  Orla **Smith**  Marlene **Santos**  Gyan **Sandhu**  Jennifer **Hodder**  Imrana **Khalid**  Dr. Paraskevi **Vlachou**  Dr. Anish **Kirpalani**  Dr. Errol **Colak**  Dr. Gevork **Mnatzakanian**  Carolyn **Trottier**  Junwu **Mu**  Roya **Akbari**  Meng **Qin**  Siu Chu **Lam**  Alexandra **Karolczyk**  Carmen **Lau**  Jessica **Fu**  Cara **Leis**  Glenn **Gabrielpillai**  Bao Truong (Leo) **Tran**  Chrystal **Douglas**  Iris **Fok**  Jieqi **Ma**  Victoria **Babysheva**  Lin **Lan** |
| **Sunnybrook Health Sciences Centre** | Dr. Robert **Fowler**  Dr. Neill **Adhikari**  Dr. Damon **Scales**  Nicole **Marinoff**  Adic **Perez**  Navjot **Kaur**  Vivekanandan **Thayalasuthan**  Sonia **Welsh** |
| **University of Alberta Hospital** | Dr. Michael **Jacka**  Dr. Sean **Bagshaw**  Nadia **Baig**  Lorena **McCoshen**  Dr. Gavin **Low**  Marina **McToal** |
| **Australia** | |
| **Gosford Hospital** | Dr. Atul **Gaur**  Katrina **Ellis**  Mary **White**  Dr. Rajiv **Rattan** |
| **Royal North Shore Hospital** | Prof. Simon **Finfer**  Associate Professor Anthony **Delaney**  Elizabeth **Yarad**  Anne **O’Connor**  Frances **Bass**  Naomi **Hammond**  Julia **Pilowsky**  Sharon **Mar**  Melissa **Owen**  Simon **Bird**  Dr. Charles **Fisher**  Linda **Thebridge**  Louise **McIntosh**  Zoe **Li**  David **Su**  Katherine **Prasad** |
| **St Vincent’s Hospital, Sydney** | Dr. Hergen **Buscher**  Claire **Reynolds**  Nerilee **Baker**  Karlee **McCann**  Elizabeth **Pluis** |
| **India** | |
| **King George’s Medical University** | Dr. Zia **Arshad**  Sachin Kumar **Srivastava**  Avinash **Singh**  Archit **Deva**  Dr. Shailesh Kumar **Singh**  Dr. Amit **Kushwaha** |
| **Medanta, The Medicity** | Dr. Yatin **Mehta**  Joby V. **George**  Dr. Chitra **Mehta**  Dr. Ashish **Kumar**  Jyoti **Chandel**  Dr. Gaurav **Kochhar**  Bhuwan **Singh** |
| **Vijaya Orthopedic and Trauma Center** | Dr. Mrityunjaya **Uppin**  Devesh **Kumar**  Anit **Singh**  Dr. Vinayak **Kabate** |
